# Supplementary material for: Seminoma and Embryonal Carcinoma Footprints Identified by Analysis of Integrated Genome-Wide Epigenetic and Expression Profiles of Germ Cell Cancer Cell Lines
Source: PLoS One. 2014 Jun 2;9(6):e98330. doi: 10.1371/journal.pone.0098330 (PMC4041891; doi:10.1371/journal.pone.0098330)
Supplement: File S1 — ZIP file containing DMRforPairs output for significant regions. Please start from the html files. (ZIP) [file pone.0098330.s008.zip › figures/39000.pdf]

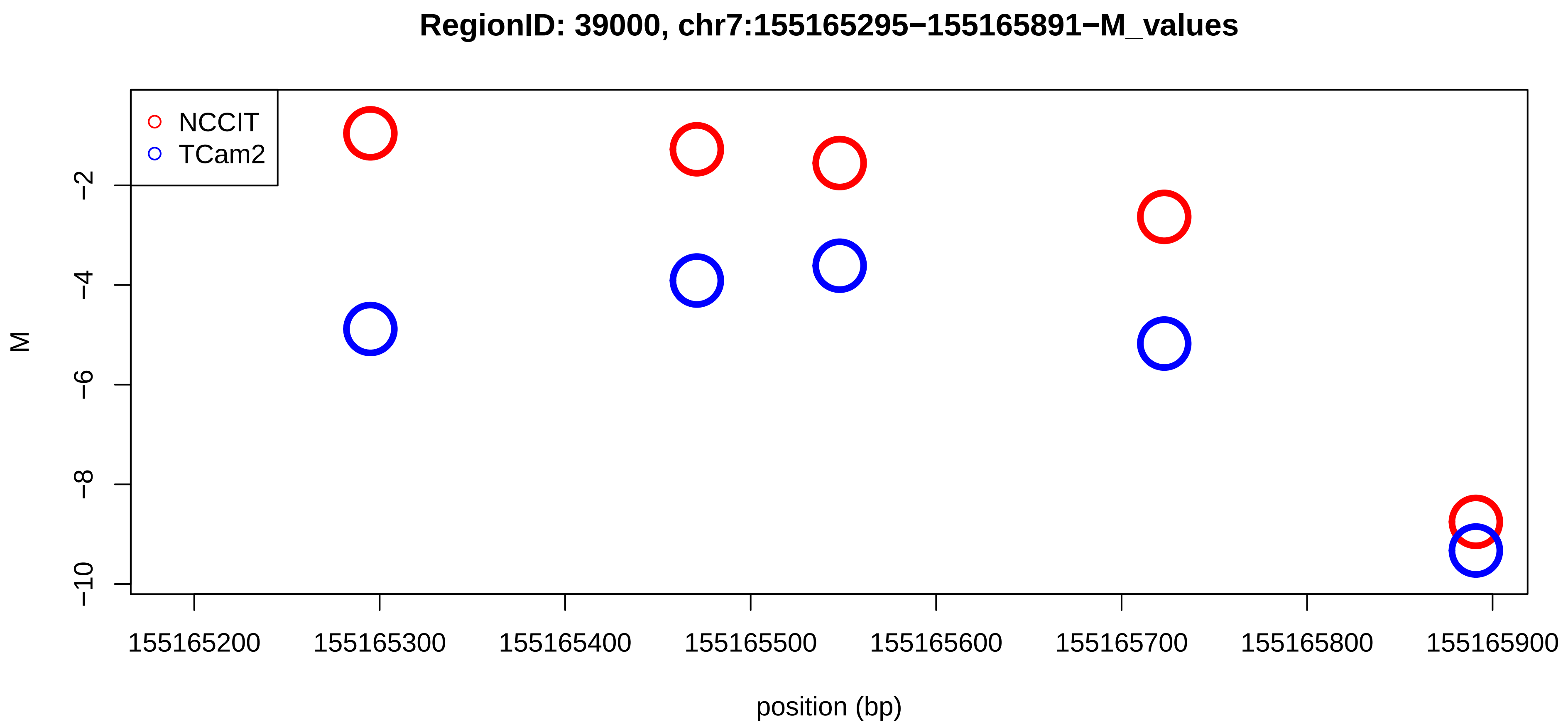
RegionID: 39000, chr7:155165295−155165891−M_values
NCCIT
TCam2
M
−2
−4
−6
−8
−10
155165200
155165300
155165400
155165500
155165600
155165700
155165800
155165900
position (bp)

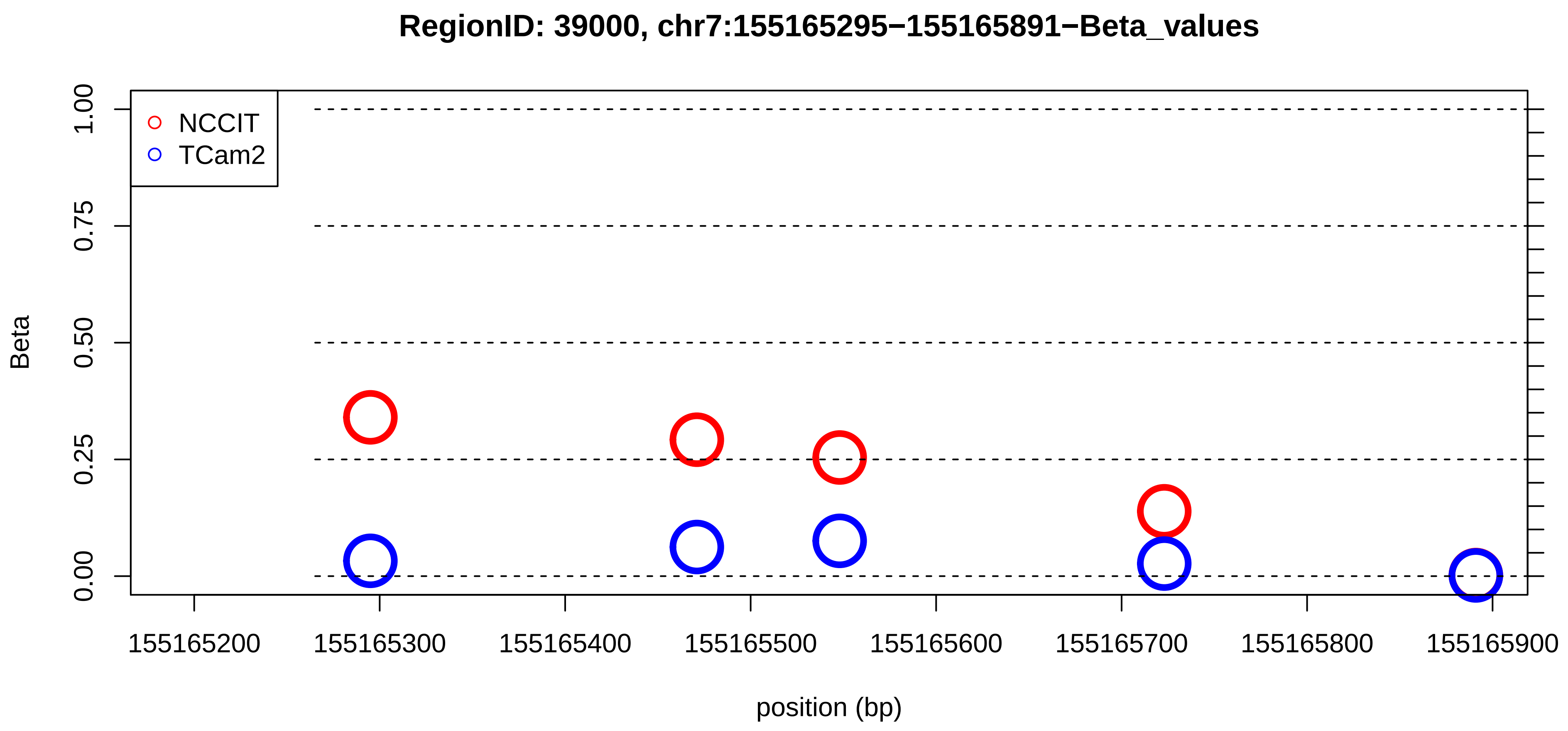
RegionID: 39000, chr7:155165295−155165891−Beta_values
NCCIT
TCam2
Beta
0.00
0.25
0.50
0.75
1.00
155165200
155165300
155165400
155165500
155165600
155165700
155165800
155165900
position (bp)
